# Correlates of the Intention to Implement a Tailored Physical Activity Intervention: Perceptions of Intermediaries

**DOI:** 10.3390/ijerph110201885

**Published:** 2014-02-10

**Authors:** Denise Peels, Aart Mudde, Catherine Bolman, Rianne Golsteijn, Hein de Vries, Lilian Lechner

**Affiliations:** 1 Department of Psychology and Educational Sciences, Open University of the Netherlands, Heerlen, P.O. Box 2960, Heerlen 6401 DL, The Netherlands; E-Mails: aart.mudde@ou.nl (A.M.); catherine.bolman@ou.nl (C.B.); rianne.golsteijn@ou.nl (R.G.); lilian.lechner@ou.nl (L.L.); 2 Department of Health Promotion, Maastricht University, Maastricht University Medical Centre, P.O. Box 616, Maastricht 6200 MD, The Netherlands; E-Mail: Hein.deVries@maastrichtuniversity.nl; 3 Care and Public Health Research Institute (Caphri), Maastricht University, Maastricht 6200 MD, The Netherlands

**Keywords:** intervention implementation, hypothesized determinants of implementation intention, tailored intervention, intervention characteristics, organisational characteristics, socio-political characteristics, intermediary characteristics

## Abstract

The public health impact of health behaviour interventions is highly dependent on large-scale implementation. Intermediaries—intervention providers—determine to a large extent whether an intervention reaches the target population, and hence its impact on public health. A cross-sectional study was performed to identify the correlates of intermediaries’ intention to implement a computer-tailored physical activity intervention. According to theory, potential correlates are intervention characteristics, organisational characteristics, socio-political characteristics and intermediary characteristics. This study investigated whether intermediary characteristics mediated the association between the intervention, organisational and socio-political characteristics and intention to implement the intervention. Results showed that intervention characteristics (*i.e.*, observability (B = 0.53; *p* = 0.006); relative advantage (B = 0.79; *p* = 0.020); complexity (B = 0.80; *p* < 0.001); compatibility (B = 0.70; *p* < 0.001)), organisational characteristics (*i.e.*, type of organization (B = 0.38; *p* = 0.002); perceived task responsibility (B = 0.66; *p* ≤ 0.001); capacity (B = 0.83; *p* < 0.001)), and the social support received by intermediary organisations (B = 0.81; *p* < 0.001) were associated with intention to implement the intervention. These factors should thus be targeted by an implementation strategy. Since self-efficacy and social norms perceived by the intermediary organisations partially mediated the effects of other variables on intention to implement the intervention (varying between 29% and 84%), these factors should be targeted to optimise the effectiveness of the implementation strategy.

## 1. Introduction

Although the health benefits of regular physical activity (PA) are well known, many people are still not sufficiently active. Evidence-based interventions to stimulate PA are therefore urgently needed. Although numerous studies on the efficacy of PA interventions have been published, few studies have addressed the preconditions for implementation of these proven interventions. This is, however, essential as the public impact of effective interventions is highly dependent on their implementation: when PA interventions are not implemented adequately in practice, they will clearly not have the intended effects. Several reports have noted this substantial gap between scientific knowledge and public health practice [1,2,3]. Within the field of health behaviour promotion, intermediaries (such as general health practitioners, nurses or municipal health counselors) are often an important link between the developers of the intervention and the target population (*i.e.*, the users). Often these intermediaries determine the final exposure of the intervention to the target population, they therefore have an essential role in the implementation process [4].

In the present study, we investigated intermediaries’ opinions about implementation of a tailored intervention to stimulate PA among people aged over fifty years. Stimulating PA in this age group is of major importance, since it reduces the risks of health problems, which often increase with age [5,6]. It also enables older adults to maintain their mobility and independence and improves their quality of life [6,7,8]. The studied intervention, named Active Plus, is a computer-tailored, theory driven and evidence-based intervention, which can be provided in a print-delivered and a Web-based format. The intervention optionally includes additional information about local PA opportunities, tailored to the user’s PA preferences (e.g., addresses of sports locations matching his or her preferences, cycling or walking routes). The intervention has proved effective in enhancing weekly minutes of PA and weekly days with sufficient PA up to six months after the start of the intervention. One year after the start of the intervention it still resulted in increased weekly days with sufficient PA, and was borderline effective (*p* = 0.071) in increasing weekly minutes of PA [9]. Although proven effective, the actual public impact of the intervention will be highly dependent on its implementation in practice. Insight into the determinants of intermediaries’ intention to implement the intervention is of importance to establish a good implementation strategy.

Intervention implementation can be defined as active, planned efforts to implement an innovation within a defined setting [10]. Active Plus can be regarded as an innovation as this type of intervention (specifically in the Web-based format) is relatively new, especially among older adults. The research framework of this study is therefore based on Rogers’s Theory of Innovations [11] and the framework of determinants of innovation processes described by Paulussen *et al.* [4]. These theories outline how the implementation might be influenced by a wide range of factors. Rogers’s Theory of Innovations [11] defines five innovation characteristics that might influence the decision to adopt or reject an innovation. The first characteristic is relative advantage, *i.e.*, the degree to which an innovation is perceived as being better than the idea it supersedes: in this instance, whether the implementation of Active Plus is perceived as being better than other (e.g., face-to-face) PA interventions.

The second characteristic is compatibility, *i.e.*, the degree to which an innovation is perceived as being consistent with the existing values, past experiences, and needs of potential users. If the intermediary (provider, practitioner) can adapt an innovation to his or her own needs, the innovation will be adopted more easily. Although Active Plus has a solid basis in several behavioural change theories which cannot be changed by the intermediary, the intermediary can adapt the intervention to his or her own needs by adding additional environmental information to the intervention (e.g., information about PA opportunities in the local area, references to other PA interventions), and an intermediary may choose to provide the intervention in a print-delivered or in a Web-based format.

The third innovation characteristic is complexity *i.e.*, the degree to which an innovation is perceived as difficult to understand and use (in other words, do intermediaries think the implementation process will be straightforward or do they anticipate difficulties e.g., with preparing the tailored advice letters).

The fourth characteristic is trialability *i.e.*, the degree to which one can experiment with an innovation on a limited basis, with minimal risks (in other words, whether intermediaries can try the intervention on a small scale without obligation and with minimal risk).

The fifth characteristic is observability; the more observable the intervention effects are to others (e.g., policymakers, users), the more willing intermediaries will be to implement the intervention (in other words, if intermediaries expect that the health of the inhabitants of their region will be visibly improved by participating in the intervention they are more likely to be willing to use it) [11].

The Rogers’s theory of Innovations [11] and the framework of Paulussen [4] both propose that the characteristics of an organisation in which the intermediary acts play an important role in the implementation process. Factors such as the size, perceived task responsibility, and capacity might influence whether an organisation is willing or able to adopt the innovation. Both theories also argue that the socio-political context—such as rules and social support from other organisations—can influence the implementation process.

A final determinant of implementation is provider or practitioner characteristics (*i.e.*, the intermediaries’ (individual or perceived organisational) attitude, subjective norms, self-efficacy to implement the intervention, and knowledge of the intervention). It has been stated that organisations will not change *i.e.*, implement an innovation unless their employees are willing to change [12]. As well as being interested in the effects of the intervention on the target population, intermediaries also want to know what the consequences of implementing the intervention will be for themselves and whether they expect themselves to be able to implement the intervention [12]. 

Whereas Rogers’s Theory of Innovations mainly provides insight into the direct influence of different factors on the implementation process, Paulussen argues that intermediary characteristics (proximal factors) might mediate the influence of intervention characteristics, organisational characteristics and the socio-political characteristics (distal factors) on the implementation process. Similar mediation processes are proposed in diffusion models based on the Integrated Change Model [13,14]. To our knowledge, no previous studies have identified the pathway, *i.e.*, the mediating factors, of hypothesized determinants of intention to implement an intervention. This knowledge, however, is important because these insights might help us to determine what factors can best be targeted by the implementation strategy.

The purpose of the present study was twofold: (1) to provide insight into the hypothesized determinants of intermediaries’ intention to implement the tailored Active Plus intervention; and (2) to establish a theoretical framework describing the association between intervention characteristics, organisational characteristics and socio-political characteristics on the one hand, and the potentially mediating intermediary-provider-characteristics on the other hand. These analyses are an important step in establishing a good implementation strategy—thus ensuring the intervention achieves maximum effect on public health—and contributing to the establishment of a good theory for the large-scale implementation of interventions by intermediaries.

## 2. Methods

Quantitative data was gathered in accordance with our research framework in order to identify facilitating and hindering factors for the implementation of the Active Plus intervention.

### 2.1. Study Population, Design and Procedure

The starting point for this study was a series of semi-structured in-depth interviews with intermediaries (the health promoters of five municipal health counselor (MHC) organisations who had previously participated in the Active Plus effectiveness study) to identify the most important concepts for each construct. The interviews provided the information needed to construct the questionnaire used in the current study. Additionally it was assessed which organisations were potential intermediaries for the implementation of this intervention in a non-experimental setting. Further content and results of these interviews are beyond the scope of this study.

**Figure 1:**
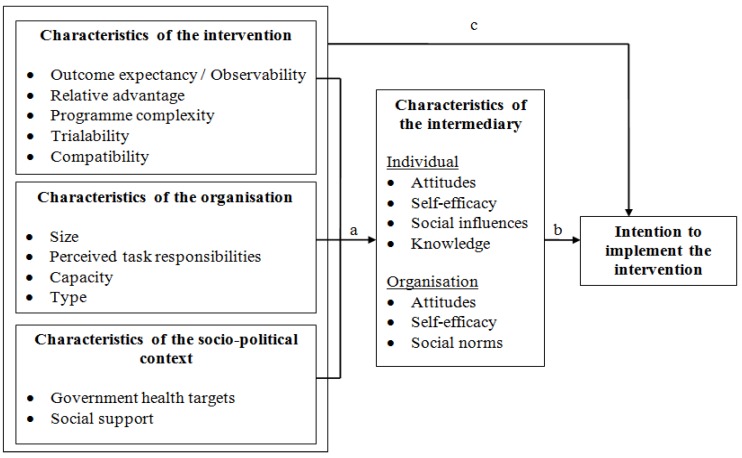
The theoretical framework.

Notes: the a-path represents the association between the distal factors and the proximal factors associated with intention; the b-path represents the association between the proximal factors and the intention;the c-path represents the direct association between the distal factors and intention; the framework is based on Rogers’s Theory of Innovations [11] and the framework of determinants of innovation processes by Paulussen *et al.* [4].

Based on information from the semi-structured interviews, guided by our research framework (as discussed in the introduction and presented graphically in Figure 1), and the implementation questionnaire used by Bessems *et al.* [15], a questionnaire was developed to measure variables relating to implementation of the Active Plus intervention. A recruitment letter including the questionnaire, and a flyer with information about the intervention, its benefits, and the cost and time needed to implement the intervention was sent to organisations which had been identified as important intermediaries by the interviews: the policy employees and health promoters of all MHCs in the Netherlands (*N =* 29); the civil servants of all the health and/or sports department of municipalities (*N* = 415) and all regional sport-service organisations (*N* = 12). 

### 2.2. Intervention

Active Plus is a computer-tailored, theory and evidence-based intervention to stimulate or maintain PA in adults aged over fifty years by targeting its psycho-social determinants [16]. The intervention can be provided in a print- or Web-based format, and optionally includes additional information about local PA opportunities and initiatives intended to positively change users’ perceptions about local opportunities [17]. 

Intervention participants receive tailored advice at three time points: (1) within two weeks of the baseline assessment; (2) two months after the baseline assessment; and (3) up to four months after baseline assessment, after filling in the second assessment. Tailored advice is based on the answers given in the previous assessment [16,18] and comprises between five and eleven pages of material, adapted according to changes in the participant’s PA behaviour and determinant scores [16,18].

The print-based intervention resulted in higher participation rates than the Web-based intervention (19% *versus* 12%) [19], but also had higher implementation costs per participant and involved more manual labour than the Web-based intervention. Providing the optional additional local information also increased implementation costs [9]. As mentioned in the introduction, the intervention has been shown to be effective in enhancing PA. Adding local information did not result in increased intervention effects [9]. 

### 2.3. Measurement Instrument

A questionnaire was developed based on our research framework, the information gathered in the in-depth interviews, and the implementation questionnaire used by Bessems *et al.* [15]. The characteristics of the intervention, the organisation, the socio-political context and the intermediary were assessed. The characteristics of the intermediary were assessed regarding their individual perceptions and regarding the perceptions as perceived by the organisation of the intermediary. The number of items used to measure a certain construct, Cronbach’s α for the construct scale, and example questions are provided in Table 1. 

**Table 1:** Measurements of characteristics of the intervention, organisation, socio-political context, intermediary, and their reliability.

Concept	Items (N)	Example Question/Statement	α
***Intervention characteristics***			
Relative advantage	15	Compared to other interventions, implementation costs for this intervention are low. *Totally disagree (1)* to *Totally agree (5)*	0.67
Trialability	2	Implementing this intervention requires large financial investments. *Totally disagree (1)* to *Totally agree (5)*	0.70
Outcome expectancy/Observability	10	By implementing this intervention, PA behaviour of people aged over 50 within our region will increase. *Totally disagree (1)* to *Totally agree (5)*	0.88
Programme complexity	4	Implementation the Active Plus intervention is uncomplicated. *Totally disagree (1)* to *Totally agree (5)*	0.69
Compatibility	4	The intervention corresponds with our targets. *Totally disagree (1)* to *Totally agree (5)*	0.78
***Organisational characteristics***			
Size	1	How many employees has your organisation?	-
Perceived task responsibility	6	It is our organisation’s responsibility to stimulate PA among people aged over 50. *Totally disagree (1)* to *Totally agree (5)*	0.73
Capacity	9	Our organisation has sufficient staff capacity to implement the intervention. *Totally disagree (1)* to *Totally agree (5)*	0.87
Type	1	In which type of organisation are you working? *Municipality/Sport-Service/MHC*	-
***Socio-political context***			
Governmental health targets	2	The Active Plus intervention corresponds to the national health targets. *Totally disagree (1)* to *Totally agree (5)*	0.73
Social support	8	I expect to get support for intervention implementation from (other) MHC’s. *Certainly not (1)* to *Yes, definitely (5)*	0.76
***Intermediary characteristics***			
*Individual*			
Attitude	7	The insecurities of new interventions are worrisome to me. *Totally disagree (1)* to *Totally agree (5)*	0.71
Self-efficacy	4	I am able to convince my colleagues of the need to implement a new intervention. *Totally disagree (1)* to *Totally agree (5)*	0.76
Social support	1	My colleagues will support me when I announce a new idea. *Totally disagree (1)* to *Totally agree (5)*	-
***Intermediary characteristics***			
Subjective norm	1	My social environment welcomes innovations. *Totally disagree (1)* to *Totally agree (5)*	-
Knowledge	5	I have sufficient knowledge about the consequences of physical inactivity among persons aged over 50. *Totally disagree (1)* to *Totally agree (5)*	0.82
*Organisation*			
Attitude	3	Promoting PA in persons aged over 50 within our region is important for our organisation. *Totally disagree (1)* to *Totally agree (5)*	0.77
Self-efficacy	4	Our organisation is able to implement this intervention. *Totally disagree (1)* to *Totally agree (5)*	0.74
Subjective norm	7	Welfare organisations think it is—*Very unimportant (1)* to *Very important (5)*—that our organisation implements this intervention	0.82
***Intention to implement Active Plus***			
	2	When the Active Plus intervention is available, I will coordinate/execute the intervention implementation within one year. *Certainly not (1)* to *Yes, definitely (5)*	0.79

Notes: α stands for the reliability of each scale. A reliability of 0.6–0.7 is acceptable. A reliability > 0.7 is good.

The outcome measure was intention to implement the intervention, assessed with two items on a five-point Likert scale from *Certainly not* (1) to *Yes, definitely* (5). 

### 2.4. Analyses

The analyses were conducted with SPSS version 21.0. Mean scale scores and standard deviations were calculated for each construct. Mediation analyses were performed to identify the hypothesized determinants of intermediaries’ intention to implement the intervention, and to identify whether the characteristics of the intervention, organisation or socio-political context were directly associated with this intention, or were mediated by intermediary characteristics. The mediation analyses consisted of four steps [20]: (1) estimating the direct association between the distal factors—*i.e.*, intervention characteristics, the organisational and the socio-political characteristics—and intention to implement the intervention (c-pathway in Figure 1); (2) estimating the association between distal factors and proximal factors *i.e.*, intermediary characteristics (a-pathway); (3) estimating the association between proximal determinants and intention to implement the intervention (b-pathway) controlling for the association with the distal factors (c’-pathway); and (4) testing the significance of the product of coefficients (a × b) by computing the associated asymmetric bias-corrected bootstrap confidence intervals. The criteria for mediation were met when the 95% confidence interval (CI) did not include zero. The proportion of the effect of the distal factors that was mediated by the proximal determinant was calculated using the following formula: [(a × b)/(c’ + (a × b))]. As recommended by MacKinnon, only significant mediation mechanisms were included in calculating the proportion being mediated since the inclusion of insignificant mediators may lead to ambiguous results [21]. All analyses were controlled for the age, gender and educational level of the intermediary. The mediation analyses were performed using the PROCESS macro for SPSS from Hayes [22]. This macro simplifies the estimation process described above by conducting all these regressions (per distal factor) with one command and also generates various additional inferential tests for indirect effects which are not available in the standard OLS regression in SPSS. 

## 3. Results

### 3.1. Characteristics of the Sample

A total of 10 regional sport-service organisations (83% response rate), 117 municipalities (28% response rate) and 19 Municipal Health Counselors (MHCs; 66% response rate) participated in this study. The majority of respondents (*n* = 146) were female (61.4%) with an average age of 44.4 years (SD = 11.87); 1.1% had a medium vocational school degree, 56.2% had a higher vocational school degree and 42.7% had a university degree. 

Of the sport-service organisations, 66.7% reported that they intended to implement the intervention, 2.9% of the municipalities and 11.1% of the MHCs intended to implement the intervention (resulting in an average of 8.4% potential adopters across all organisations). The mean scale scores and standard deviations for intervention characteristics, the organisational characteristics, socio-political characteristics and the intermediary characteristics, and the percentage of respondents having a positive score on that scale are presented in Table 2. For most constructs, the majority of the respondents had a positive score. Only regarding the relative advantage of the intervention, the programme complexity and the trialability of the intervention, the majority of the respondents had a negative score.

### 3.2. Factors Associated with the Intentions to Implement the Intervention

As shown in Table 3 (c-pathway); almost all ‘distal’ factors had a direct association with intention to implement the intervention. Of the intervention characteristics; observability (*B* = 0.53; *p* = 0.006); relative advantage (*B* = 0.79; *p* = 0.020); complexity (*B* = 0.80; *p* = 0.000) and compatibility (*B* = 0.70; *p* = 0.000) were directly associated with intention to implement the intervention. The positive B-values (i.e. the unstandardized coefficients) show that a more positive opinion of the intermediary regarding these intervention characteristics is associated with a higher intention to implement the intervention in practice. The higher the B-value, the stronger the positive association is between the intervention characteristic and the intermediaries’ intention. Of the organisational characteristics; type of organisation (*B* = 0.38; *p* = 0.002); perceived task responsibility (*B* = 0.66; *p* = 0.000) and capacity (*B* = 0.83; *p* = 0.000) were directly positively associated with intention to implement the intervention. Size was the only assessed organisational characteristic that was not associated with intention to implement the intervention. In terms of socio-political characteristics; the amount of social support the intermediary organisation receives (*B* = 0.81; *p* = 0.000) was positively associated with intention to implement the intervention; government health targets had a borderline association with intention to implement the intervention (*B* = 0.23; *p* = 0.097). 

Of the proximal factors (see Table 4), only self-efficacy of the organisation and the social norms perceived by the organisation were positively associated with intention to implement the intervention. None of the individual intermediary characteristics was associated with intention to implement. A borderline significant association between organisational attitude and intention to implement the intervention was found. The *B*s and *p*-values for the proximal factors relating to intention to implement the intervention differ for each analysis because the numbers of participants in each analysis varied as a result of missing data on distal correlates.

**Table 2:** Mean scores and standard deviation (SD) for the characteristics of the intervention, the organization, the socio-political context and of the intermediary.

Potential Determinants	Number of Observations (*N*)	Mean	SD	% of Respondents Having a Positive Score
***Intervention characteristics***				
Outcome expectancy/Observability	142	3.45	0.43	78.8
Relative advantage	136	3.09	0.26	45.9
Programme complexity	137	3.26	0.54	44.1
Trialability	135	2.76	0.59	20.5
Compatibility	140	3.54	0.62	74.7
***Organisational characteristics***				
Size (amount of employees)	126	268.89	373.61	
Perceived task responsibility	145	3.59	0.53	84.2
Capacity	137	2.85	0.70	35.6
*Socio-political context*				
Governmental health targets	140	3.83	0.61	82.9
Social support	132	3.28	0.44	67.1
***Intermediary characteristics***				
*Individual*				
Attitude	145	3.71	0.47	91.1
Self-efficacy	145	3.49	0.53	80.1
Subjective norm	146	3.69	0.64	69.2
Social support	146	3.67	0.64	67.1
Knowledge	143	3.56	0.63	80.8
***Organisation***				
Attitude	146	3.84	0.60	90.4
Self-efficacy	139	3.22	0.66	61.0
Subjective norm	133	3.52	0.46	82.2
Intention to implement the intervention	139	2.30	0.92	8.4

Notes: factors are assessed on a scale from (1) to (5), in which 5 is the most positive outcome. A score above 3 was indicated as having a positive score.

**Table 3:** Association between distal factors and proximal factors (a-path), and the association between distal factors and intention (with (c’-path) and without correction (c-path) for the proximal factors.

Potential Determinants		Intermediairy Characteristics (a-path)								Intention to Implementation	
		Individual					Organisational				
	*N*	Attitude (SE)	Self-efficacy (SE)	Social norm (SE)	Social support (SE)	Knowledge (SE)	Attitude (SE)	Self-efficacy (SE)	Social norm (SE)	c’-path (SE)	c-path (SE)
***Intervention characteristics***											
Observability	100	0.33 (0.09) ***	0.36 (0.11) **	0.31 (0.13) *	0.42 (0.13) **	0.16 (0.13)	0.32 (0.12) **	0.32 (0.14) *	0.33 (0.09) ***	0.11 (0.17)	0.53 (0.19) **
Relative advantage	121	0.52 (0.17) **	0.54 (0.20) **	0.71 (0.23) **	0.71 (0.23) **	0.03 (0.24)	−0.01 (0.22)	0.57 (0.24) *	0.78 (0.15) ***	0.11 (0.33)	0.79 (0.34) *
Complexity	120	0.18 (0.08) *	0.24 (0.09) *	0.21 (0.11) ^‡^	0.10 (0.11)	0.13 (0.11)	0.24 (0.10) *	0.71 (0.10) ***	0.21 (0.08) **	0.32 (0.16) ^‡^	0.80 (0.15) ***
Trialability	120	0.07 (0.07)	0.07 (0.08)	0.14 (0.10)	−0.01 (0.10)	−0.20 (0.10) *	−0.05 (0.09)	0.27 (0.10) **	−0.03 (0.07)	−0.03 (0.13)	0.06 (0.14)
Compatibility	120	0.29 (0.06) ***	0.20 (0.08) *	0.18 (0.09) ^‡^	0.19 (0.09) *	0.24 (0.09) *	0.47 (0.08) ***	0.39 (0.09) ***	0.35 (0.06) ***	0.26 (0.14) ^‡^	0.70 (0.12) ***
***Organisational characteristics***											
Type	121	0.09 (0.06)	0.05 (0.07)	0.09 (0.09)	0.10 (0.09)	0.26 (0.08) **	0.12 (0.08)	0.09 (0.09)	0.01 (0.06)	0.29 (0.10) **	0.38 (0.12) **
Size	105	−0.00 (0.00)	0.00 (0.00)	0.00 (0.00)	0.00 (0.00)	0.00 (0.00)	−0.00 (0.00)	0.00 (0.00)	0.00 (0.00) *	−0.00 (0.00)	−0.00 (0.00)
Responsibility	121	0.41 (0.07) ***	0.31 (0.09) ***	0.18 (0.11) ^‡^	0.22 (0.11) *	0.43 (0.10) ***	0.50 (0.09) ***	0.49 (0.10) ***	0.31 (0.07) ***	0.09 (0.16)	0.66 (0.14) ***
Capacity	121	0.24 (0.07) ***	0.30 (0.08) ***	0.23 (0.09) *	0.25 (0.09) **	0.39 (0.09) ***	0.44 (0.08) ***	0.66 (0.08) ***	0.25 (0.06) ***	0.38 (0.16) *	0.83 (0.11) ***
***Socio-political***											
Health targets	120	0.23 (0.07) ***	0.02 (0.08)	0.07 (0.10)	−0.00 (0.10)	0.30 (0.09) **	0.19 (0.09) *	0.08 (0.10)	0.22 (0.06) **	−0.03 (0.13)	0.23 (0.14) ^‡^
Social support	119	0.54 (0.09) ***	0.48 (0.11) ***	0.40 (0.14) **	0.48 (0.14) ***	0.31 (0.14) *	0.23 (0.13) ^‡^	0.27 (0.15) ^‡^	0.53 (0.09) ***	0.50 (0.20) *	0.81 (0.19) ***

Notes: * *p* < 0.05; ** *p* < 0.01; *** *p* < 0.001; ^‡^ *p* < 0.10; Values presented are the unstandardized coefficients; SE = Standard error; N = Number of participants included per analysis.

**Table 4:** Association between proximal factors and intention to implement the intervention (b-path).

Potential Determinants	Intention to Implement the Intervention (b-path)										
	Intervention Characteristics					Organisational Characteristics				Socio-cognitive Characteristics	
	Observability (SE)	Relative advantage (SE)	Complexity (SE)	Trialability (SE)	Compatibility (SE)	Type (SE)	Size (SE)	Responsibility (SE)	Capacity (SE)	Health targets (SE)	Social support (SE)
***Individual intermediary characteristics***											
Attitude	−0.15 (0.20)	−0.17 (0.20)	−0.16 (0.20)	−0.13 (0.20)	−0.15 (0.20)	−0.16 (0.19)	−0.06 (0.22)	−0.18 (0.21)	−0.10 (0.20)	−0.15 (0.20)	−0.29 (0.21)
Self-efficacy	0.04 (0.16)	0.06 (0.16)	0.03 (0.16)	0.06 (0.16)	0.09 (0.16)	0.07 (0.16)	0.05 (0.17)	0.06 (0.16)	0.02 (0.16)	0.05 (0.17)	0.03 (0.16)
Social norm	0.03 (0.16)	0.05 (0.16)	−0.01 (0.16)	0.06 (0.16)	0.04 (0.16)	0.04 (0.15)	0.11 (0.17)	0.05 (0.16)	0.01 (0.16)	0.06 (0.16)	0.06 (0.16)
Social support	−0.11 (0.17)	−0.14 (0.17)	−0.07 (0.17)	−0.12 (0.17)	−0.11 (0.17)	−0.16 (0.16)	−0.09 (0.18)	−0.13 (0.17)	−0.11 (0.16)	−0.15 (0.17)	−0.19 (0.17)
Knowledge	0.16 (0.12)	0.16 (0.12)	0.14 (0.12)	0.13 (0.12)	0.13 (0.12)	0.07 (0.12)	0.11 (0.13)	0.14 (0.12)	0.06 (0.12)	0.16 (0.12)	0.14 (0.12)
***Organisational intermediary characteristics***											
Attitude	0.26 (0.14) ^‡^	0.29 (0.14) *	0.29 (0.14) *	0.27 (0.14) ^‡^	0.14 (0.15)	0.26 (0.13) ^‡^	0.17 (0.15)	0.26 (0.14) ^‡^	0.20 (0.14)	0.28 (0.14) *	0.31 (0.14) *
Self-efficacy	0.53 (0.11) ***	0.53 (0.12) ***	0.40 (0.13) **	0.52 (0.12) ***	0.51 (0.11) ***	0.52 (0.11) ***	0.54 (0.12) ***	0.52 (0.12) ***	0.37 (0.13) **	0.53 (0.12) ***	0.54 (0.11) ***
Social norm	0.65 (0.18) ***	0.64 (0.18) ***	0.62 (0.17) ***	0.63 (0.17) ***	0.54 (0.19) **	0.68 (0.17) ***	0.50 (0.20) *	0.64 (0.18) ***	0.56 (0.17) **	0.67 (0.18) ***	0.48 (0.18) *

Notes: * *p* < 0.05; ** *p* < 0.01; *** *p* < 0.001; ^‡^ *p* < 0.10; Note: proximal factors are presented in the left column of the table. Distal characteristics presented at the second/third row of the table are only presented since they slightly influence the findings because the numbers of participants in each analysis varied as a result of missing data on distal correlates; Number of participants included in the analyses per determinant are similar in each path-way, and can be found in Table 3; Values presented are the unstandardized coefficients; SE = Standard error.

**Table 5:** Mediating mechanism (ME) of intermediary characteristics on the intention to implement the intervention.

Potential Deteminants	*Individual Intermediary Characteristics*					*Organisational Intermediary Characteristics*					
	Attitude	Self-efficacy	Social norm	Social support	Knowledge	Attitude		Self-efficacy		Social norm	
	ab-path (95% CI)	ab-path (95% CI)	ab-path (95% CI)	ab-path (95% CI)	ab-path (95% CI)	ab-path (95% CI)	% ME	ab-path (95% CI)	% ME	ab-path (95% CI)	% ME
***Intervention characteristics***											
Observability	−0.05 (−0.26–0.07)	0.02 (−0.12–0.17)	0.01 (−0.12-0.15)	−0.05 (−0.24–0.09)	0.03 (−0.01–0.13)	** 0.08 (0.00–0.26)**	**43.8**	**0.17 (0.01–0.38)**	**61.4**	** 0.21 (0.10–0.40)**	**66.7**
Relative advantage	−0.09 (−0.35–0.10)	0.03 (−0.12–0.34)	0.03 (−0.21–0.34)	−0.10 (−0.50–0.09)	0.00 (−0.05–0.11)	−0.00 (−0.17–0.10)	**-**	**0.30 (0.10–0.62)**	**73.2**	** 0.50 (0.21–0.88)**	**81.7**
Complexity	−0.03 (−0.18–0.02)	0.01 (−0.08–0.09)	−0.00 (−0.09–0.08)	−0.01 (−0.09–0.03)	0.02 (−0.01–0.11)	0.07 (−0.00–0.22)	**-**	**0.29 (0.10–0.52)**	**47.2**	** 0.13 (0.04–0.31)**	**29.2**
Trialability	−0.01 (−0.08–0.01)	0.00 (−0.02–0.06)	0.01 (−0.03–0.12)	0.00 (−0.03–0.06)	−0.03 (−0.12–0.01)	−0.01 (−0.12–0.03)	**-**	**0.14 (0.05–0.29)**	**84.2**	−0.02 (−0.14–0.06)	**-**
Compatibility	−0.04 (−0.19–0.06)	0.02 (−0.04–0.13)	0.01 (−0.05–0.10)	−0.02 (−0.16–0.03)	0.03 (−0.02–0.11)	0.07 (−0.07–0.23)	**-**	**0.20 (0.10–0.34)**	**43.8**	** 0.19 (0.06–0.37)**	**42.8**
***Organisational characteristics***											
Type	−0.01 (−0.10–0.02)	0.00 (−0.02–0.06)	0.00 (−0.03–0.06)	−0.02 (−0.11–0.01)	0.02 (−0.04–0.09)	0.03 (−0.00–0.11)	**-**	0.05 (−0.02–0.13)	**-**	0.01 (−0.09–0.13)	**-**
Size	0.00 (0.00–0.00)	0.00 (0.00–0.00)	0.00 (0.00–0.00)	0.00 (−0.00–0.00)	0.00 (0.00–0.00)	0.00 (−0.00–0.00)	**-**	0.00 (−0.00–0.00)	**-**	−0.00 (−0.00–0.00)	**-**
Responsibility	−0.07 (−0.27–0.08)	0.02 (−0.09–0.16)	0.01 (−0.06–0.10)	−0.03 (−0.15–0.04)	0.06 (−0.04–0.20)	0.13 (−0.01–0.31)	**-**	**0.25 (0.13–0.43)**	**73.5**	** 0.20 (0.08–0.39)**	**68.5**
Capacity	−0.02 (−0.13–0.06)	0.01 (−0.11–0.11)	0.00 (−0.09–0.10)	−0.03 (−0.18–0.04)	0.02 (−0.07–0.12)	0.09 (−0.03–0.24)	**-**	**0.24 (−0.08–0.45)**	**39.3**	** 0.14 (0.05–0.27)**	**27.1**
***Socio-political***											
Health targets	−0.03 (−0.15–0.05)	0.00 (−0.03–0.06)	0.00 (−0.02–0.07)	0.00 (−0.04–0.04)	0.05 (−0.02–0.15)	0.05 (−0.00–0.19)	**-**	0.04 (−0.07–0.17)	**-**	** 0.15 (0.04–0.31)**	**123.7**
Social support	−0.16 (−0.43–0.03)	0.01 (−0.15–0.19)	0.03 (−0.10–0.21)	−0.09 (−0.37–0.06)	0.04 (−0.01–0.19)	0.07 (−0.01–0.24)	**-**	**0.14 (0.02–0.33)**		** 0.26 (0.02–0.52)**	**34.0**

Notes: Values presented are the unstandardized coefficients; Number of participants included in the analyses per determinant are similar in each path-way, and can be found in Table 3; Bold a × b-paths reflect a significant mediator (*i.e.*, the CI does not include zero); CI = Confidence interval; The “%ME-column” is only presented when a significant mediation mechanism was found for that proximal factor.

### 3.3. Direct versus Indirect Associations

Table 5 shows the bias-corrected bootstrap confidence intervals (CI) of the product of the a-pathway multiplied with the b-pathway. When the 95%-CI does not include zero, a significant mediation effect was found. The percentage of this mediating mechanism (calculated as described in paragraph 2.4) is presented in Table 5 as well. As shown in Table 5, organisational attitude mediated 43.8% of the association between the observability of the intervention and intention to implement the intervention. This indicates that the observability of the intervention both has a direct association (see paragraph 3.2), as well as an indirect association (through the organisational’s attitude) with the intermediaries’ intention to implement the intervention. The self-efficacy of the organisation partially mediated the association between all intervention characteristics and the intention (varying between 43.8% and 84.2% of mediation). The social norms of the organisation also partially mediated the association between the intervention characteristics (except the trialability), and intention to implement the intervention (varying between 29.2% and 81.7% mediation). Furthermore, the association between organisational perceived task responsibility and intention to implement the intervention was mediated for 73.5% by the self-efficacy of the organization, and for 68.5% by the social norm of the organization. The association between the organisational capacity and intention to implement the intervention was mediated for 39.3% by the self-efficacy of the organization, and for 27.1% by the social norm of the organization. Furthermore, the organisation’s social norms fully mediated the association between government health targets and intention to implement the intervention.

## 4. Discussion and Conclusions

The purpose of the present study was to provide insight into the factors that are relevant to the implementation by intermediaries of a tailored PA intervention for adults aged over fifty years, and to provide insight into the association between the perceived intervention characteristics, organisational characteristics and the socio-political factors influencing intention to implement on the one hand, and the potentially mediating intermediary characteristics on the other hand. These insights are important as it is known that many interventions of proven effectiveness are not fully implemented outside an experimental context [1,2,3]. The results of this study can be used to develop a good strategy for large-scale implementation of tailored PA interventions. 

Most characteristics of the intervention, the organisation and the socio-political context had both a direct and an indirect association with intermediaries’ intention to implement the intervention. The trialability of the intervention and the size of the intermediary organisation were the only intervention characteristics not associated with intention to implement the intervention. The association between all remaining factors—apart from organisational type—and intention to implement the intervention was partially mediated by the self-efficacy of the intermediary organisation to implement the intervention, and the social norms of the intermediary organisation. The mediating effect of these intermediary characteristics is in line with the framework outlined by Paulussen [4] and the Integrated Model for Change [13,14]. However, whilst these models focus on the individual perceptions of the intermediary, the current study has shown that implementation process was not mediated by the individual characteristics of the intermediary, but by the self-efficacy and social norms of his or her intermediary organisation as perceived by the intermediary. To our knowledge, this is the only study to date which has formally identified the mediating mechanisms of the intermediary characteristics on the association between the intervention characteristics, organisational characteristics, socio-political factors and intention to implement a PA intervention.

The association between the intention to implement an innovation and the intervention characteristics, self-efficacy and social support identified in the current study is in line with a literature review by Fleuren *et al.* [23], and the association with self-efficacy and compatibility is in line with a review by Durlak and DuPre [24]. A study of factors underlying motivation of primary health care professionals to implement a lifestyle intervention also identified social support, intervention compatibility and perceived relative advantage as factors associated with intention to implement the intervention [25]. The review by Fleuren *et al.* also showed that organisational size did not influence motivation to implement the intervention [23].

Based on the results of the current study, several adaptations can be proposed for existing models trying to explain the implementation process. First of all, in contrast to the framework of Paulussen [4] and diffusion models based on the I-Change Model [13,14], the effect of the intervention characteristics, organisational characteristics, and socio-political characteristics was not fully mediated by the characteristics of the intermediary. Most factors were also directly associated with the intention to implement the intervention. Furthermore, current results showed that the effect of the more distal factors was not mediated by the individual characteristics of the intermediary. Instead, the self-efficacy and the social norm of the organisation, as perceived by the intermediary mediated the effect of the distal factors on the intention to implement the intervention. However, since this is the first study formally identifying the pathway (*i.e.*, the mediating factors) of hypothesized determinants of intention to implement an intervention, more research is needed to establish a theoretical framework identifying the relations between these factors. Furthermore, the existing models focus on the (mediating) characteristics of the end-user of the intervention, instead of the characteristics of the intermediary (*i.e.*, the provider of the interventions) as studied in the current paper. More research is necessary to identify whether pathways explaining the intermediaries’ intention to implement the intervention differ from the pathways explaining the intention of the end-user to implement the intervention.

### 4.1. Strengths and Limitations

To our knowledge, this is the first study that has identified the factors associated with intermediaries’ intention to implement a tailored PA intervention, and the first study that has identified the potential pathway for these factors. One of the strengths of the current study is that it provides insight into the associated factors in advance of the decision to implement the intervention and therefore also includes the opinions of intermediaries who might decide not to implement the intervention. Studies are often performed with intermediaries who already have a strong commitment to implement the intervention, or have implemented the intervention already [25,26,27,28] and thus often only include first adopter categories (*i.e.*, innovators or early adopters according to Rogers [11]). The current study also included later adopter categories (*i.e.*, the late majority adopters and laggards), and the results of this study might therefore be more generalizable to the population of potential intermediaries than previous studies. In order to reach the majority of the potential intermediaries for intervention implementation, these insights are of major importance. 

A limitation of the current study is that all the analyses relied on self-reported data, which might be biased towards socially desirable responses. There also might be some divergence between organisations who claim a positive intention to implement the intervention, and those who will actually implement the intervention. Furthermore, response might be biased by the personal intentions for implementation of the respondent (e.g., it could be that their personal preference is transferred to their opinion of what the intention of the organisation should be). Performing multilevel analyses including more employees per organisation would be recommendable for future research. 

The current study was not able to provide insight into the sustainability of the intervention implementation, which is also important in determining the impact of the intervention on public health. Additional analyses should provide insight into the gap between the intention to implement and actual implementation, and factors that influence the sustainability of the intervention. 

A further limitation of the current study is that owing to the small sample sizes for MHCs and the sport-service organisations—high response rates notwithstanding—intermediaries were analysed as a single group. However, due to the current task divergence between the different intermediary organisations, the predictors of implementation intention may differ between organisational types. In the Netherlands, the municipalities receive a certain governmental budget that can be used to implement health behaviour interventions or to stimulate PA behaviour; municipalities have the responsibility to allocate these budgets, but most often they will not execute the intervention implementation themselves. Sport-service organisations, on the other hand, do not have an own budget for intervention implementation but are a more practical organisation with more possibilities to execute the intervention implementation. The MHC’s often advice the municipalities where to allocate their budgets, and can be an important link between the municipalities and the sport-service organisations. This might explain the large variations in the intention to implement the intervention between the different intermediary organisations as well. Furthermore, providing the intermediaries more actual insight in the intervention itself (which was not done in the current study) might have changed certain perceived intervention characteristics (e.g., the perceived complexity) which might have increased the intention to implement the intervention. A final limitation of this study is that because of its cross-sectional design, causal conclusions could not be drawn. Although the proposed causal pathways were based on a theoretical model [4], longitudinal research is needed to determine whether mediation really occurs. Despite these limitations, this study provides very useful insights that should be used to develop a good implementation strategy for tailored interventions and thus ensure they have maximum impact on public health.

### 4.2. Implications for Practice

In conclusion, the results of this study show that the intervention characteristics (except trialability), organisational characteristics (except size) and the social support received by the intermediary organisation are all directly associated with intention to implement the intervention, and should thus be included and dealt with in the development of an implementation strategy. To optimise the effectiveness of the implementation strategy, the self-efficacy of the intermediaries’ organisation to implement the intervention should be increased, and the social norms perceived by intermediary organisations should be targeted, since these characteristics partially mediate the association between the other factors and intention to implement the intervention. 

Especially the relative advantage and the complexity of the intervention should be targeted in the implementation materials, as both concepts were associated with the intention to implemented the intervention, but received a low score by the majority of the respondents. To target the relative advantage, implementation materials should specify and represent graphically the effects of the intervention, and its advantages over other health behaviour interventions. Perceptions about the complexity of the intervention should be targeted explicitly by illustrating the straightforward structure and procedures of the intervention. To emphasise the compatibility of the intervention, materials should specify how the intervention procedures map to current working processes and the targets of the intermediary organisation. 

Because the type of organisation and its capacity were directly related to intention to implement the intervention, the task divergence of different types of intermediary organisations should be taken into account when developing these implementation materials. The perceived task responsibility of intermediary organisations should be targeted to stimulate their intention to implement the intervention (e.g., by providing examples of how the intervention might help the organisation to reach its targets and highlighting the ways in which the intervention aligns with the organisation’s responsibilities). User-centred development, *i.e.*, collaboration between the researchers and practitioners, of implementation materials is recommended to ensure the best possible fit between intervention procedures and the working processes of the intermediary organisation. Previous studies have shown that a user-centred design also results in better program sustainability [3,4,24]. 

The association of the intervention characteristics, organisational characteristics and the social support with the intention to implement the intervention is partially mediated by the self-efficacy of the intermediary organisation to implement the intervention and the social norm perceived by the intermediary organisation, so the effect of the implementation strategy could be optimised by targeting the self-efficacy and the social norm as well. Organisational self-efficacy might be increased by use of methods such as guided practice, enactive mastery (*i.e.*, demonstrating to intermediaries how the intervention works and how it can be implemented in practice, and practicing these actions together), verbal persuasion and self-monitoring (*i.e.*, feedback on the positive effects of the intervention) as part of implementation training [29]. Furthermore, implementation training should include methods such as enhancing network linkages in order to stimulate social support and the social norm for intervention implementation (*i.e.*, training network members to provide support and to mobilise and maintain their networks, providing a webpage where intermediaries of the intervention can discuss experiences and problems). 

Development of an implementation strategy based on these results should provide a better basis for successful and sustainable intervention implementation and thereby maximise the public health effect of the intervention. Future research should reveal whether the usage of systematically developed implementation materials would result in different intentions to implement a proven effective health behaviour intervention (e.g., comparing two groups, one group taking into account the strategies mentioned in the current section *versus* one group who receives practice as usual). Overall, in the current study the first step was made in gaining insight in the factors associated with the intention to implement a proven effective health behaviour intervention in practice.
